# Development of a Cell Culture Model for Inducible SARS-CoV-2 Replication

**DOI:** 10.3390/v16050708

**Published:** 2024-04-29

**Authors:** Xiaoyan Wang, Yuanfei Zhu, Qiong Wu, Nan Jiang, Youhua Xie, Qiang Deng

**Affiliations:** 1 Key Laboratory of Medical Molecular Virology (MOE/NHC/CAMS), School of Basic Medical Sciences, Shanghai Institute of Infectious Disease and Biosecurity, Fudan University, Shanghai 200032, China; 18111010063@fudan.edu.cn (X.W.); zhuyuanfei@fudan.edu.cn (Y.Z.); 21111010078@m.fudan.edu.cn (Q.W.); 22111010066@m.fudan.edu.cn (N.J.); 2 Shanghai Frontiers Science Center of Pathogenic Microorganisms and Infection, Fudan University, Shanghai 200032, China

**Keywords:** severe acute respiratory syndrome coronavirus 2, replicon, inducible model

## Abstract

Severe acute respiratory syndrome coronavirus 2 (SARS-CoV-2) induces direct cytopathic effects, complicating the establishment of low-cytotoxicity cell culture models for studying its replication. We initially developed a DNA vector-based replicon system utilizing the CMV promoter to generate a recombinant viral genome bearing reporter genes. However, this system frequently resulted in drug resistance and cytotoxicity, impeding model establishment. Herein, we present a novel cell culture model with SARS-CoV-2 replication induced by Cre/*LoxP*-mediated DNA recombination. An engineered SARS-CoV-2 transcription unit was subcloned into a bacterial artificial chromosome (BAC) vector. To enhance biosafety, the viral spike protein gene was deleted, and the nucleocapsid gene was replaced with a reporter gene. An exogenous sequence was inserted within NSP1 as a modulatory cassette that is removable after Cre/*LoxP*-mediated DNA recombination and subsequent RNA splicing. Using the PiggyBac transposon strategy, the transcription unit was integrated into host cell chromatin, yielding a stable cell line capable of inducing recombinant SARS-CoV-2 RNA replication. The model exhibited sensitivity to the potential antivirals forsythoside A and verteporfin. An innovative inducible SARS-CoV-2 replicon cell model was introduced to further explore the replication and pathogenesis of the virus and facilitate screening and assessment of anti-SARS-CoV-2 therapeutics.

## 1. Introduction

Severe acute respiratory syndrome coronavirus 2 (SARS-CoV-2), which is highly contagious, has led to a global pandemic with catastrophic consequences for humans [1]. Despite the emergency approval of oral therapeutic drugs such as Paxlovid and Molnupiravir providing partial relief for severely ill patients, the absence of a specific antiviral therapeutic drug underscores the need for novel treatment strategies [2,3]. Addressing this challenge requires a deeper understanding of the molecular mechanisms underlying viral infection, replication, and virus–host interactions. Viral replicons, consisting of viral DNA or RNA molecules capable of autonomous replication within host cells, have emerged as indispensable tools in molecular virology [4]. These replicons are usually engineered with various genetic manipulations, including the deletion of structural genes, insertion or replacement of reporter genes, and screening markers, for distinct purposes.

The RNA viral replicon system is a useful tool for virology research, involving the in vitro transcription of DNA vectors to generate recombinant viral genomic RNA. Subsequent introduction of viral RNA into cultured cells using electro-transduction facilitates autonomous replication of viral RNA. Several studies have reported the construction of SARS-CoV-2 model systems by manipulating its RNA [5,6,7,8]. However, the complexities associated with in vitro transcription and RNA transfection experiments pose significant challenges. To address this, some studies have employed DNA vectors encoding viral genomes for cell transfection that utilize CMV promoters to initiate viral RNA transcription and achieve autonomous RNA replication using nucleocytoplasmic transfer [9,10,11,12]. Despite these advancements, recombinant viral reporters often exhibit background transcriptional activity within the nucleus, along with inherent splicing mechanisms involved in nuclear-transcribed RNA. Efforts to identify suitable cell models based on existing replicon systems, including those pursued by our research team, have faced significant challenges. The replication of SARS-CoV-2 may potentially induce toxic effects, such as the inhibition of host translation mechanisms and the stimulation of reactive oxygen species (ROS) responses, thereby impeding long-term cell viability [13,14,15].

To overcome these limitations, we innovatively established an inducible SARS-CoV-2 replicon cell model in this study. This model may increase the viral replication efficiency while circumvent the concomitant cytotoxicity, thereby providing a more stable, efficient, and convenient experimental system for analysis of SARS-CoV-2 replication and pathogenesis. Additionally, this model holds promise for wide application in screening and evaluating anti-SARS-CoV-2 drugs.

## 2. Materials and Methods

### 2.1. Cell Culture

Vero E6, Huh7.5, and BHK-21 cells were maintained in Dulbecco’s modified Eagle’s medium (DMEM) supplemented with 10% fetal bovine serum (FBS) (DMEM and FBS; Biological Industries, Kibbutz Beit Haemek, Israel) and 1% penicillin-streptomycin. GlutaMAX™ (Cat. No. 35050061; Thermo Fisher Scientific, Waltham, MA, USA) was added to Huh7.5 cells at a final concentration of 1× in the medium. Cells were cultured at 37 °C and a pH of 7.2–7.4 in an atmosphere containing 5% CO_2_ for optimal growth.

### 2.2. Construction of Plasmids

The pcDNA3.1-N plasmid was constructed using PCR to obtain the N gene product from c, and homologous recombination was performed using the Seamlessing mix (Cat. No. D7010S; Beyotime Biotechnology, Shanghai, China) according to the manufacturer’s instructions. The pCDH-Cre plasmid was constructed in a previous study [16]. The pCDH-N-IRES-Cre plasmid was constructed with PCR using pcDNA3.1-N, pCDH-Cre, and pLVX-IRES-Puro (Cat. No. P0249; Miaoling Biology, Wuhan, China) as templates to obtain the N, IRES, and Cre fragments. SeamLess mix was used to obtain the target plasmid with pCDH as the vector.

### 2.3. Assembly of Rep-S-EGFP/NLuc

The cDNA sequences (5′UTR, M, E, N, ORF3a/b, ORF7a/b, ORF10, 3′UTR) were obtained from the genome of SARS-CoV-2 strain nCoV-SH01 (GenBank: MT121215.1) in a previous study [17]. The coding sequence of the Spike (S) gene was replaced with either T2A-EGFP or T2A-IgK-Nanoluciferase (NLuc), while retaining the transcriptional regulatory sequences. Using the overlap PCR method, various sequences, including CMV, HDV RZ (synthesized gene by GENEWIZ), BGH pA, and SV40 pA, were cloned into the pBeloBAC11 vector. This method employs Primer Star GLX (Cat. No. R051A; Takara, Ohtsu, Japan). PCR was performed to amplify the desired fragments, which are then cloned into the vector using homologous recombination. This resulted in the successful construction of intermediate plasmids pBAC11-5′UTR-EGFP-3′UTR and pBAC11-5′UTR-NLuc-3′UTR. The ORF1ab gene of SARS-CoV-2 was fragmented into 4 segments in the pCC1Bac-ORF1ab plasmid (Cat. No. PA11799; GENEWIZ, South Plainfield, NJ, USA), each with 40 bp homologous sequences at their ends. Fragments F1, F2, F3, and F4 were obtained using PCR amplification. These fragments were then assembled into the pBAC11-2-5′UTR-EGFP-3′UTR and pBAC11-2-5′UTR-NLuc-3′UTR vectors using Gibson Assembly HiFi Mix (Cat. No. A46627; Thermo Fisher Scientific). Following the previously established protocol for the pBeloBAC11 (Cat. No. P0908; Miaoling Biology, Wuhan, China) vector, the noninfectious replicon plasmids Rep-S-EGFP and Rep-S-NLuc were successfully obtained. In Rep-S-NLuc, a portion of the N gene coding sequence was deleted, and the NLuc coding sequence was inserted in the place of the S gene, resulting in the construction of Rep-N-NLuc. The replicon plasmids were extracted using the NucleoBond BAC 100 (Cat. No. 740579; Macherey-Nagel, Düren, Germany) maxi prep kit for large-construct plasmids.

### 2.4. Establishment of the iRep-N-NLuc Plasmid

The pCC1Bac-ORF1ab plasmid was linearized using KasI and BstBI restriction endonucleases to obtain an approximately 20 Kb ORF1ab linear fragment designated as Fragment 1. Fragment 2 (BstBI-S-ORF3-E-M-ORF6-ORF7-ORF8-ORF9-N-NLuc-ORF10-3′UTR-HDV-RZ-core-Insulator-3′PiggyBacTR) was amplified using fusion PCR. The vector backbone was amplified through PCR using the pBeloBAC11 vector as a template, and the resulting fragment was termed Fragment 3. Fragment 4 (5′PiggyBacTR-core-insulator-CMV-5′UTR-NSP1-5′intron-*LoxP*1-P2A-Blasticidin-SV40 polyA-BGH polyA-*LoxP*2-3′intron-NSP1-KasI) was amplified using fusion PCR as well. Fragments F1, F2, F3, and F4 were then assembled using Gibson assembly to generate the recombinant plasmid iRep-N-NLuc.

### 2.5. Transposition of iRep-N-NLuc Plasmid into BHK-21 Cell Chromatin to Establish a Cell Culture Model

The iRep-N-NLuc and Super PiggyBac transposase (Cat. No. P0179; Miaoling Biology) plasmid were cotransfected into BHK-21 cells at a 5:1 plasmid-to-transposase ratio using Lipofectamine 3000 (Cat. No. L300150; Thermo Fisher Scientific), following the manufacturer’s protocol. At 24 h post-transfection, the culture medium was replaced with fresh medium containing 10 μg/mL blasticidin (Cat. No. A1113903; Thermo Fisher Scientific). Selection with blasticidin was maintained by replacing the complete medium with fresh medium containing 10 μg/mL blasticidin every 12 h.

### 2.6. Plasmid Transfection

Preparation of replication-competent DNA mixtures: In a 12-well plate, after replacing the culture medium of cells at 80–90% confluence with fresh complete culture medium, 2 μg of the plasmid (per well) was mixed with 50 μL Opti-MEM (Cat. No. 31985070; Thermo Fisher Scientific) and mixed, followed by the addition of 4 μL P3000. The solution was mixed well and incubated at 25 °C for 2 min.

Dilution of transfection reagent: Subsequently, 4 μL Lipofectamine 3000 solution was mixed with 50 μL Opti-MEM and added to the mixture in (1). After mixing, the mixture was incubated at 25 °C for 10 min, added to each well in the cell culture plate, and gently shaken. The medium in each well was replaced after 12 h of transfection.

### 2.7. RT-qPCR for sgRNAs Detection

Total RNA was extracted using Trizol (Cat. No. 15596026CN; Thermo Fisher Scientific) and reverse transcribed into cDNA using the FastKing-RT SuperMix solution (Cat. No. KR118; TIANGEN, Beijing, China) for RT-qPCR. Subsequently, RT-qPCR was performed using the SuperReal PreMix Plus (Cat. No. FP205-02; TIANGEN) with specific primers on a Light-Cycler^®^ 480 Instrument II (Roche, Basel, Switzerland). The primer sequences for the detection of the N gene subgenomic region were as follows: N-sg-F: 5′-TTCCCAGGTAACAAACCAACCA-3′, N-sg-R: 5′-GGTTACTGCCAGTTGAATCTGAG-3′, N-NLuc-sg-F: 5′-TCCCAGGTAACAAACCAACCA-3′, N-NLuc-sg-R: 5′-CACACTCCCTGTTCAAGGAC-3′, hamster β-actin-F: 5′-CAGCACCATGAAGATCAAGATCATT-3′, and β-actin-R: 5′-CGGACTCATCGTACTCCTGCTT-3′.

### 2.8. Luciferase Reporter Gene Assay

The assay was performed according to the manufacturer’s instructions. Briefly, for IgK-NLuc, which carries a secretion signal, the supernatant was directly collected for detection using the Nano-Glo Luciferase Assay System (Cat. No. N1150; Promega, Madison, WI, USA) 24 h after transfection. For NLuc (without a secretion signal), cells were washed with PBS and lysed with 65 µL (for a 48-well plate) of lysis buffer containing a protease inhibitor cocktail for 30 min on a shaking platform at 100 rpm. Then, 10 µL of sample was mixed with 10 µL of substrate working solution and measured using a GloMax20/20 luminometer (Promega, Madison, WI, USA). The data were recorded. Subsequently, the Renilla detection protocol was performed by adding 10 µL Stop&Glo (Cat. No. E1910; Promega) to measure Renilla luminescence, and the data were record. Statistical analysis of the data was conducted.

### 2.9. Western Blotting

The assays were performed as previously described [18]. Briefly, cell samples were washed thrice with PBS and lysed with 2 × SDS loading buffer containing β-mercaptoethanol. The lysates were boiled at 100 °C for 10 min. Protein samples were then separated using 12% SDS-PAGE gel electrophoresis and transferred to PVDF membranes. Membranes were blocked with 5% nonfat milk in TBST at 25 °C for 2 h. After washing thrice with TBST, membranes were incubated with the respective primary antibodies at 4 °C overnight. Subsequently, membranes were incubated with secondary antibodies, and the signals were detected using ECL (Cat. No. 180-5001; Tanon, Shanghai, China). The antibodies used in this study were as follows: anti-GAPDH (Cat. No. 60004-1-Ig; Proteintech, Wuhan, China), anti-Lamin B1 (Cat. No. 66095-1-Ig; Proteintech), anti-nucleocapsid (generated in a previous study [17]), goat anti-rabbit IgG H&L-HRP (Cat. No. ab205718; Abcam, Cambridge, UK), and goat anti-mouse IgG H&L-HRP (Cat. No. ab205719; Abcam).

### 2.10. Compounds

In total, 56 monomeric compounds of traditional Chinese medicine were purchased from Selleck (Houston, TX, USA). Remdesivir (GS-5734) and nirmatrelvir (PF-07321332) were purchased from MedChemExpress (South Brunswick, NJ, USA). Verteporfin was purchased from TargetMol Chemicals, Inc. (Boston, MA, USA).

### 2.11. Statistical Analysis

All statistical analyses and graphs generated in this study were performed using GraphPad Prism 9 (GraphPad Software, La Jolla, CA, USA). Data are presented as the mean ± standard error of the mean (SEM). Statistical analysis between two groups was performed using Student’s *t*-test. All half maximal inhibitory concentration (IC_50_) curve data were fitted after normalization. Statistical significance was defined as follows: ns indicates no statistical difference, * indicates *p* < 0.05, ** indicates *p* < 0.01, *** indicates *p* < 0.001, and **** indicates *p* < 0.0001. *p* < 0.05 was set as the significance threshold.

## 3. Results

### 3.1. Construction and Validation of an Infection-Deficient SARS-CoV-2 Replicon Model

**Figure 1:**
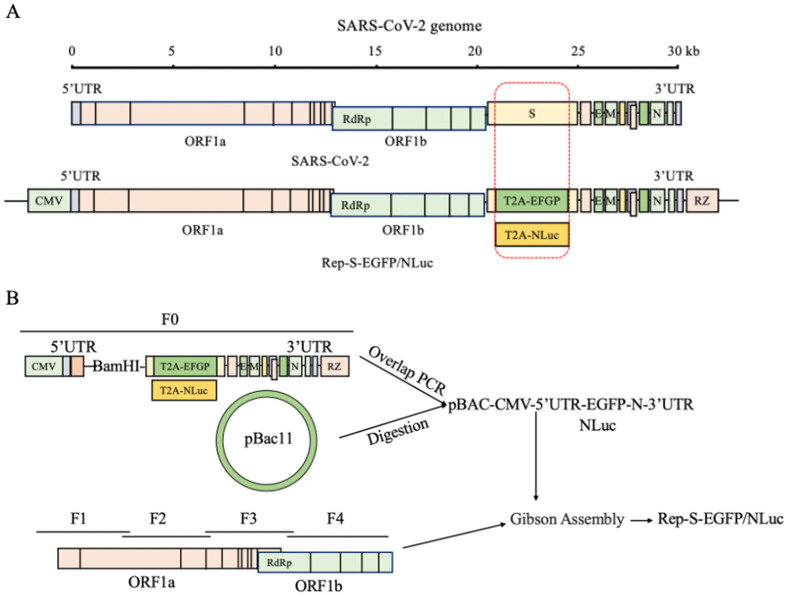
Design of the Rep-S-EGFP/NLuc replicon. (**A**) Design of the SARS-CoV-2 viral genome structure and replicon model. We selectively excised a portion of the S gene coding sequence while preserving its transcriptional regulatory sequence (TRS) and inserted the “self-cleaving peptide” T2A-linked EGFP or IgK secretion signal peptide-tagged NLuc sequences, allowing NLuc or EGFP to be expressed freely. The CMV promoter and hepatitis D virus anti-ribozyme sequence (HDV RZ) were introduced to the 5′ and 3′ ends of the SARS-CoV-2 genome, respectively, to construct the transcription unit. (**B**) Flowchart of the SARS-CoV-2 recombinant replicon assembly strategy. Utilizing a bacterial artificial chromosome (pBAC) as the vector, the pBAC-CMV-5′UTR-EGFP/NLuc-N-3′UTR precursor clone was generated using fusion PCR. This precursor clone was subsequently digested with restriction enzymes and ligated with ORF1ab segments (F1/F2/F3/F4) harboring homologous arms, culminating in the assembly of the replicon clone plasmid Rep-S-EGFP/NLuc using Gibson assembly.

To develop a biosafety replicon model for observing and quantifying replicon replication, we initially deleted a portion (aa36–1252) of the SARS-CoV-2 S protein DNA-coding sequence. This segment was replaced with T2A-EGFP or T2A-IgK-NLuc while maintaining critical transcription regulatory and RNA secondary structure sequences essential for viral RNA transcription and replication. The CMV promoter and HDV RZ sequences were inserted into the 5′ and 3′ ends of the SARS-CoV-2 viral DNA-coding sequence, respectively, forming a transcriptional unit (Figure 1A). Using a low-copy bacterial artificial chromosome BAC as a vector, we constructed a non-infectious SARS-CoV-2 reporter plasmid, R-S-EGFP, or R-S-NLuc (Figure 1B).

**Figure 2:**
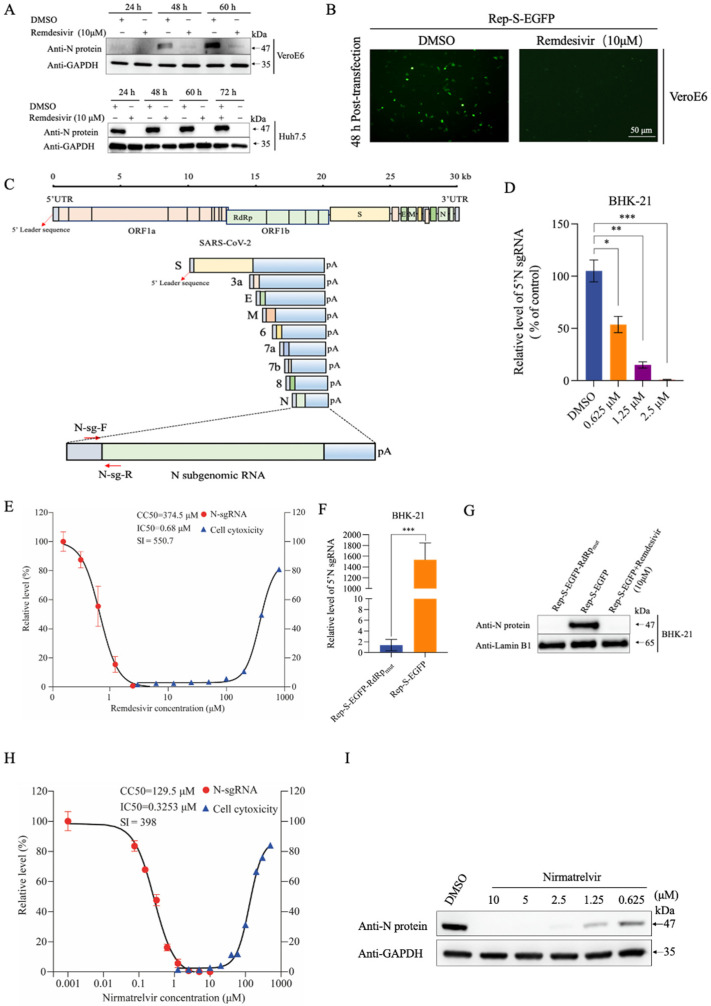
Rep-S-EGFP replication in transfected cells. (**A**) Changes in nucleocapsid (N) protein levels in Rep-S-EGFP-transfected cells and the effect of remdesivir on its expression. The Rep-S-EGFP replicon plasmid was transfected into VeroE6 and Huh7.5 cells. After 6 h, 10 μM remdesivir was added. Cell lysates were collected at 24, 48, 60, and 72 h post-transfection for western blotting analysis. (**B**) At 48 h post-transfection, VeroE6 cells as depicted in (**A**) were imaged under a fluorescence microscope. Representative images from three independent experiments are shown. (**C**) Schematic representation of qRT-PCR primers N-sg-F and N-sg-R targeting N gene sgRNAs. The replication of SARS-CoV-2 generates numerous subgenomic RNAs, as indicated in the diagram. (**D**) Rep-S-EGFP replicon-transfected BHK-21 cells were treated with remdesivir at indicated concentrations for 36 h. N gene sgRNA was quantified using qRT-RCR with specific primers. Each group included 3 biological replicates. * indicates *p* < 0.05, ** indicates *p* < 0.01, *** indicates *p* < 0.001. (**E**) The Rep-S-EGFP replicon plasmid-transfected BHK-21 cells were treated with different concentrations of remdesivir for 36 h. N sgRNA was determined as depicted in (**D**). Cytotoxicity was evaluated using the CCK-8 assay. Cells mock-treated with DMSO were used as control. Selectivity index (value of CC_50_/IC_50_, SI). (**F**) Differential expression of progeny N sgRNAs between the replicons Rep-S-EGFP and Rep-S-EGFP-RdRp_mut_. Data were normalized to the Rep-S-EGFP-RdRp_mut_ control. *** indicates *p* < 0.001. (**G**) Comparison of N protein levels in Rep-S-EGFP- or Rep-S-EGFP-RdRp_mut_-transfected BHK-21 cells. Treated with remdesivir for 36 h after transfection for 6 h. Cell samples were collected and analyzed using western blotting. (**H**) Rep-S-EGFP replicon-transfected BHK-21 cells were treated with nirmatrelvir at indicated concentrations for 36 h. The inhibitory effect and cytotoxicity of nirmatrelvir on Rep-S-EGFP were investigated as depicted in (**E**). (**I**) The N protein expression in nirmatrelvir-treated cells, as described in (**H**), was determined using western blotting.

Our study revealed that Rep-S-EGFP efficiently expressed the nucleocapsid (N) protein in VeroE6 and Huh7.5 cells in a time-dependent manner, with significant suppression observed upon treatment with remdesivir (Figure 2A). Similarly, there was a marked reduction in EGFP fluorescence intensity in the remdesivir-treated cells compared with that of mock-treated control (Figure 2B). These data suggest that remdesivir has a potent inhibitory activity against Rep-S-EGFP. We also noticed faint fluorescence intensity in the remdesivir-treated group, presumably attributed to incomplete repression following remdesivir treatment or background noise independent of viral replication. Employing specific qRT-PCR primers targeting the N gene sgRNAs containing the 5′ leader sequence, a hallmark exclusively produced during viral replication, further supported the replicative capacity of Rep-S-EGFP (Figure 2C). Remdesivir inhibited the expression of N sgRNAs of Rep-S-EGFP in BHK-21 cells in a concentration-dependent manner (Figure 2D,E). Consistent with previous studies [19], the IC_50_ of remdesivir for N sgRNAs was estimated to be 0.68 μM, with a half-maximal cytotoxic concentration (CC_50_) of approximately 374.5 μM (Figure 2E). We recently reported that protoporphyrin IX is an entry inhibitor against SARS-CoV-2 [17]. However, protoporphyrin IX did not inhibit the Rep-S-EGFP-driven viral replication as shown BHK-21 cells (Figure S1). These results demonstrated that the Rep-S-EGFP replicon replicates intracellularly to produce viral genomic and subgenomic RNA, with cryptic background signals representing a minor fraction. Notably, for unknown reasons, we observed that cryptic signals gradually accumulated over time with prolonged viral replication.

Further validation of the RNA-dependent RNA polymerase (RdRp)-dependent replication of Rep-S-EGFP was achieved by constructing a replication-defective replicon, Rep-S-EGFP-RdRp_mut_, wherein two Asp (D) (aa760–761) at the critical catalytic activity center of RdRp (NSP12) were mutated to two Ala (A) residues [20]. The Rep-S-EGFP-RdRp_mut_ group exhibited significantly reduced levels of progeny sgRNAs and N protein expression compared with those in the Rep-S-EGFP group (Figure 2F,G), indicating the loss of RdRp enzymatic activity in the mutant. We also determined dose–response curves for the currently approved therapeutic drug nirmatrelvir with the Rep-S-EGFP model. As expected, nirmatrelvir exhibited concentration-dependent inhibition of viral replication (Figure 2H,I).

Moreover, we found that proprietary compounds targeting the viral protease 3CL markedly suppressed the levels of N protein in Rep-S-EGFPs (Figure S2A–C), further confirming the functional replication of the Rep-S-EGFP replicon system within transfected cells. Collectively, these results illustrated that Rep-S-EGFP undergoes RdRp-dependent replication in host cells, generating viral sgRNAs that facilitate translation into structural proteins. However, further attempts to establish stable cell lines based on a replicon construct with the EGFP reporter gene fused in-frame with a blasticidin resistance gene were unsuccessful, likely due to potential cytotoxicity associated with SARS-CoV-2 replication.

### 3.2. Optimizing Reporter Sensitivity in the SARS-CoV-2 Replicon Model

**Figure 3:**
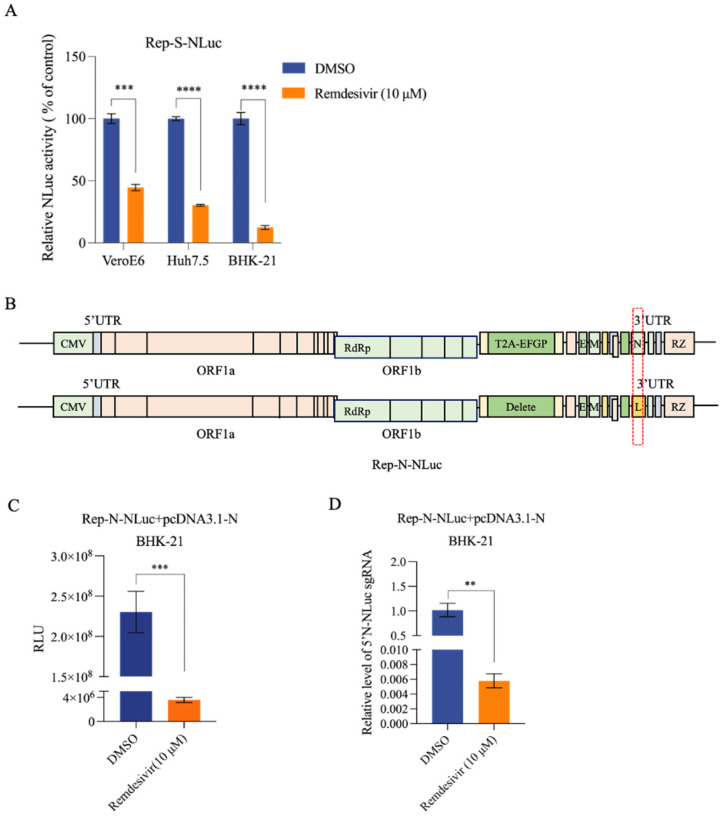
Impact of remdesivir on reporter genes in Rep-S-NLuc and optimized Rep-N-NLuc replicons. (**A**) Inhibitory effect of remdesivir on Rep-S-NLuc NLuc activity. The Rep-S-NLuc plasmid was co-transfected with a Renilla luciferase-expressing plasmid into VeroE6, Huh7.5, and BHK-21 cells. After a 6-hour transfection, 10 μM remdesivir was added. Cell lysates were collected at 36 h post-transfection for NLuc activity analysis. Each treatment group included three biological replicates. Data were normalized to that of Renilla and analyzed for differential expression, with the DMSO group serving as the control. Results are presented as the mean ± SEM. *** indicates *p* < 0.001, **** indicates *p* < 0.0001. (**B**) Optimization of the reporter gene position for the Rep-N-NLuc replicon plasmid. The N gene fragment was deleted while retaining its upstream transcription regulatory sequence (TRS). The non-secreted NLuc gene was inserted into the deleted location to rescue viral replication using N gene complementation. (**C**) Remdesivir significantly inhibited the NLuc activity (relative luminescence units, RLU) of the Rep-N-NLuc replicon. The Rep-N-NLuc replicon plasmid, pcDNA3.1-N, and Renilla luciferase-expressing plasmid were co-transfected into BHK-21 cells. The deep blue bar indicates the DMSO-treated MOCK group, while the orange bar represents the group treated with 10 μM remdesivir.Each treatment group included three biological replicates. *** indicates *p* < 0.001. (**D**) Remdesivir significantly inhibited the generation of N-NLuc sgRNAs in Rep-N-NLuc. qRT-PCR detection was performed using the samples from (**C**). N-NLuc sgRNA was detected using special primers. ** indicates *p* < 0.01.

Based on the NLuc activity, to our surprise, the inhibition efficiency of remdesivir (10 μM) against Rep-S-NLuc varied from 60% to 90% depending on the different cell types (Figure 3A). Given the observation that remdesivir almost completely abolished Rep-S-EGFP–derived N gene expression (Figure 2A,B,D), the Rep-S-NLuc system did not meet our expectations, presumably due to increased nonspecific signals independent of viral replication. Although the inhibitory effect was more pronounced in BHK-21 cells, there was only about a 10-fold reduction upon remdesivir treatment (Figure 3A). As compared with the observed Rep-S-EGFP activity, the NLuc reporter system did not meet our expectations, presumably due to increased nonspecific signals independent of viral replication. To enhance NLuc reporter sensitivity, we performed additional optimizations. Specifically, we excised a portion of the coding sequence of the N gene, known for its abundant expression, and inserted the reporter gene sequence (Rep-N-NLuc) (Figure 3B). Remarkably, in BHK-21 cells co-expressing Rep-N-NLuc and N protein, remdesivir treatment resulted in an up to 100-fold reduction in the NLuc activity (Figure 3C). Remdesivir treatment significantly suppressed the expression of N-NLuc sgRNAs in Rep-N-NLuc BHK-21 cells (Figure 3D). These findings suggested that N-protein successfully rescued Rep-N-NLuc replication, which is highly sensitive to remdesivir treatment.

### 3.3. Development of an Inducible Replicon System Expressing a Recombinant SARS-CoV-2 Genome

**Figure 4:**
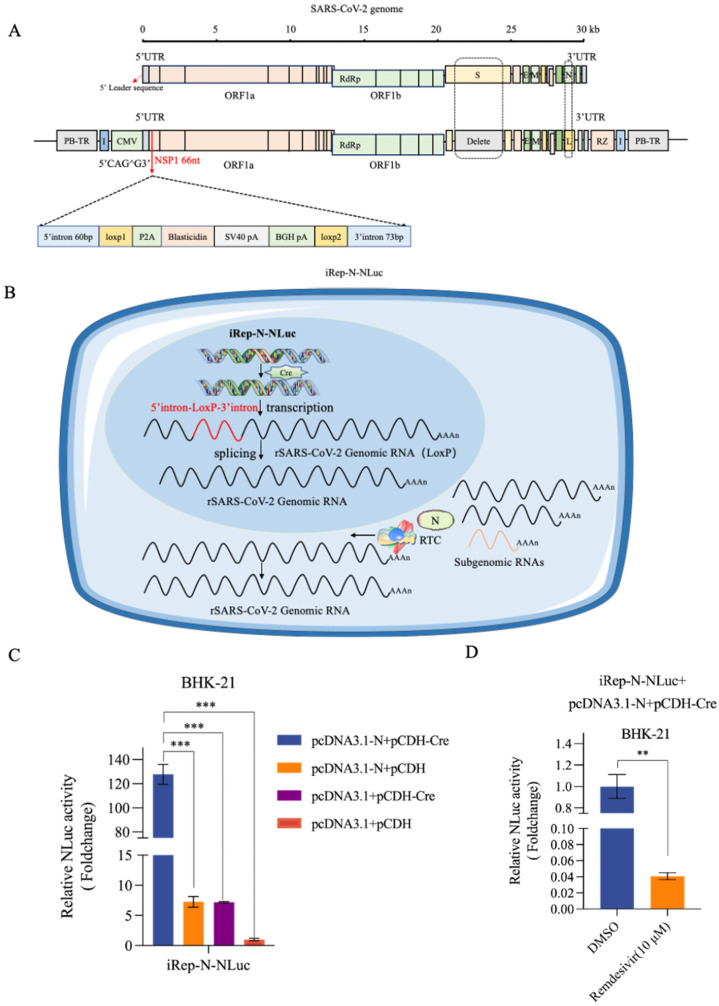
An inducible SARS-CoV-2 replicon system co-transfected with N-protein rescued viral replication upon Cre/*loxP*-mediated site-specific recombination. (**A**) Structure of the recombinant SARS-CoV-2 replicon (iRep-N-NLuc). A transcription stop cassette integrated between nt 66 and 67 (i.e., CAG66^G67; ^ indicates the potential exon/exon boundary) of the NSP1 gene. The inserted sequence consists of the indicated elements in order. PiggyBac 5′ TR and chicken β-globin insulators are inserted at the 5′ end of the CMV promoter and at the 3′ end of the HDV RZ region, respectively, flanking the transcription unit. “L” stands for NLuc. (**B**) A schema of the intracellular process of inducible iRep-N-NLuc replication in the cell model. In the absence of Cre recombinase, the CMV promoter only drives the expression of the blasticidin resistance gene, preventing SARS-CoV-2 replication, facilitating the selection of stably integrated cell lines. Following Cre recombinase induction in replicon cell lines, site-specific recombination removes the transcription stop cassette at the DNA level. The CMV promoter then initiates transcription of the viral genome. RNA splicing mechanisms within the nucleus eliminate the remaining single *LoxP* site. The cell nucleus is depicted in blue, while the cytoplasm is shown in light blue. (**C**) BHK-21 cells were co-transfected with the iRep-N-NLuc replicon and indicated plasmids plus a Renilla luciferase normalization plasmid. The fold change in NLuc activity was normalized to that of the Renilla luciferase activity. *** indicates *p* < 0.001. (**D**) BHK-21 cells were co-transfected with iRep-N-NLuc replicon, pCDNA3.1-N, and pCDH-Cre. At 6 h after transfection, cells were treated with 10 μM remdesivir for an additional 24 h and subjected to analysis of NLuc activity. Cells mock-treated with DMSO were used as a control. ** indicates *p* < 0.01.

SARS-CoV-2 replication causes prominent cellular stress and is thought to be potentially cytopathic. To address this challenge, we developed a stably integrated, inducible replicon cell model. The replicon iRep-N-NLuc utilized a preserved transcriptional module of Rep-N-NLuc and a transcription stop cassette strategically inserted within a specific locus of the NSP1 gene. This insertion comprised a 5′ intron donor sequence, a *LoxP*1 site, a blasticidin resistance gene coding sequence, two transcription termination signals for SV40 polyA and BGH polyA, a *LoxP*2 site, and a 3′ intron branching site/acceptor sequence, with both *LoxP* sites oriented in the same direction. Chicken β-globin insulator and PiggyBac transposable element sequences were inserted on both sides flanking the transcriptional unit, facilitating integration into host cell chromatin using a “cut-and-paste” mechanism mediated by PiggyBac transposase (Figure 4A).

In the absence of Cre induction, the CMV promoter controlled the expression of the blasticidin resistance gene, terminated at the polyadenylation signals. Its expression allowed for the selection of stably integrated cell lines. In the cell nucleus, Cre-mediated site-specific recombination resulted in the excision of the stop cassette, leaving only a *loxP*-chimeric intron in the replicon DNA sequence. At the RNA level, the exogenous insert was functionally removed from viral transcripts with RNA splicing (Figure 4B). The processed recombinant viral RNA was then transported to the cytoplasm with the viral replication rescued by the co-expression of the N protein. Technically, the intronic splicing was of major importance for the inducibility of the replicon. Hence, we designed two distinct insertion sites for the exogenous sequence (Figure S3A). Notably, transfection assays demonstrated efficient replication of both iRep-N-NLuc-CAGG and iRep-N-NLuc-AAGG in BHK-21 cells upon co-transfection with N protein and recombinase Cre, as indicated by the robust NLuc activity (Figure 4C). Furthermore, NLuc production was significantly suppressed by remdesivir, highlighting the hyper-sensitivity of the inducible replicon system (Figure 4D). As iRep-N-NLuc-CAGG showed higher replicability than that of iRep-N-NLuc-AAGG (Figure S3B,C), we chose the former to establish a stable integration-inducible replication system for further studies.

### 3.4. Development of a Cell-Based Inducible SARS-CoV-2 Replicon

**Figure 5:**
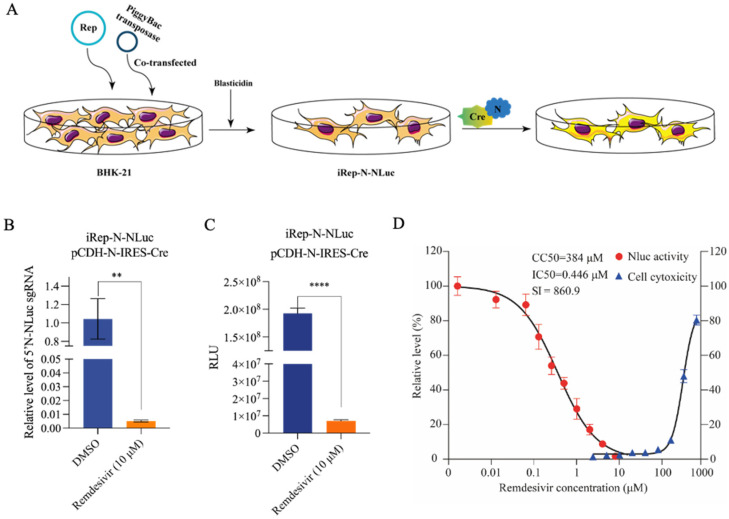
Generation of an inducible stable cell line producing a recombinant SARS-CoV-2 replicon. (**A**) Schematic illustration of the procedure to generate a cell-based SARS-CoV-2 replicon model. (**B**,**C**) The iRep-N-NLuc cell line was transfected with a bicistronic IRES vector (pCDH-N-IRES-Cre). Cells were either treated with 10 μM remdesivir or mock treated with DMSO. The recombinant N-NLuc sgRNAs (**B**) and NLuc activity (**C**) were determined at 24 h post-transfection. Error bars indicate the mean ± SEM from three independent experiments. ** indicates *p* < 0.01, **** indicates *p* < 0.0001. (**D**) pCDH-N-IRES-Cre-transfected iRep-N-NLuc cells were treated with different concentrations of remdesivir for 24 h. NLuc activity was determined. Cytotoxicity was evaluated using the CCK-8 assay. Data were expressed as the percentage of the mock-treated controls. Error bars indicate the mean ± SEM from three independent experiments.

To establish a stably integrated, inducible SARS-CoV2 replicon system, we cotransfected BHK-21 cells with the inducible replicon (iRep-N-NLuc-CAGG) plasmid and a super PiggyBac transposase expression plasmid. After blasticidin selection (10 μg/mL) for 3 d, we obtained a mixed pool of blasticidin resistant cells (Figure 5A). These cells appeared to be similar to the parental BHK-21 cells in morphology and growth dynamics.

For the convenience of the experiment, we constructed a pCDH-N-IRES-Cre plasmid that enabled the simultaneous co-expression of N protein and Cre recombinase within the same cell. Notably, we found that transfection with pCDH-N-IRES-Cre resulted in remarkable production of the N-NLuc–specific sgRNAs and robust NLuc activity in the cell-based replicon model (Figure 5B,C). In addition, remdesivir exhibited potent antiviral efficacy in this model, with an IC_50_ of approximately 0.446 μM, similar to the result observed in Rep-S-EGFP transfected cells (Figure 5D).

### 3.5. Application of the SARS-CoV-2 Replicon Cell Model for Antiviral Screening and Evaluation

**Figure 6:**
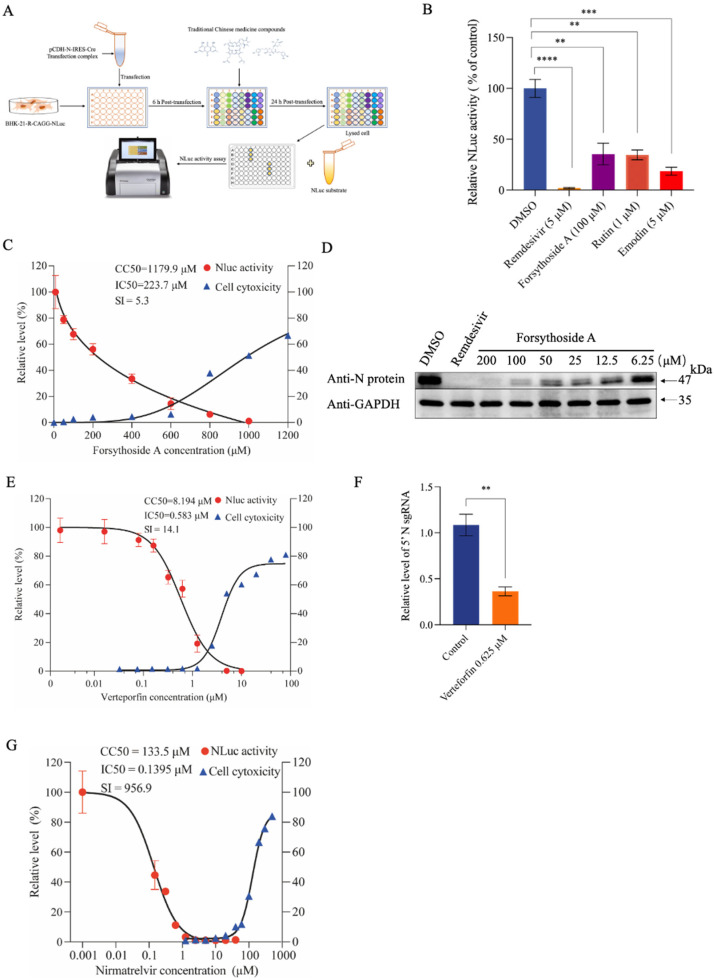
Antiviral drug screening and validation based on the inducible SARS-CoV-2 replicon cell model. (**A**) Schematic illustration of the procedure for antiviral screening based on the iRep-N-NLuc cells model. (**B**) Evaluation of the antiviral activity of candidate compounds in the iRep-N-NLuc-cells model. Cells were transfected with pCDH-N-IRES-Cre and treated with candidate compounds at indicated doses. The NLuc activity was determined at 24 h post-transfection and were expressed as a percentage of the values measured in DMSO-treated cells. Error bars indicate the mean ± SEM from three independent experiments. ** indicates *p* < 0.01, *** indicates *p* < 0.001, **** indicates *p* < 0.0001. (**C**) iRep-N-NLuc cells were transfected with pCDH-N-IRES-Cre, followed by the treatment with forsythiaside A at indicated doses for 24 h. NLuc activity was determined as depicted in (**B**). Cell viability was measured using the CCK-8 assay. (**D**) BHK-21 cells were transfected with Rep-S-EGFP and treated with forsythiaside A at indicated doses. After 48 h, N protein expression was determined using western blotting. (**E**) Rep-N-NLuc-transfected cells were treated with varying concentrations of verteporfin for 24 h. NLuc activity was measured and expressed as a percentage of the values measured in mock-treated cells. Cell cytotoxicity and CC_50_ were assessed using the CCK-8 assay. (**F**) Rep-S-GFP-transfected cells were treated with verteporfin for 36 h. N sgRNA was determined using qRT-PCR. Data are presented as the mean ± SEM from three independent experiments. ** indicates *p* < 0.01. (**G**) iRep-N-NLuc cells were transfected with pCDH-N-IRES-Cre for 6 h and subsequently treated with varying concentrations of nirmatrelvir. NLuc activity was determined as depicted in (**B**). The cytotoxicity assay is the same as the CCK8 assay in Figure 2G.

We next used the replicon cell culture model to screen 56 traditional Chinese medicine monomers recommended by physicians from the Shanghai University of Traditional Chinese Medicine mainly based on the traditional pharmacologic characteristics of these compounds. Using this small-scale screen (Figure 6A), we identified several compounds, including forsythoside A, emodin, and rutin that possessed potential antiviral activity against SARS-CoV-2 replication (Figure 6B).

Forsythoside is a major component of the traditional Chinese medicine Lianhua Qingwen that has been clinically used for treating SARS-CoV-2 infections. The dose–response curve for forsythoside A demonstrated a significant inhibition of NLuc reporter gene activity in the cell-based iRep-N-NLuc model, with an IC_50_ value of 223.7 μM. The CC_50_ of forsythoside A was estimated to be 1179.9 μM (Figure 6C). Interestingly, the antiviral effect of forsythoside A was even more marked in BHK-21 cells transfected with Rep-S-EGFP, as indicated by the level of SARS-CoV-2 N protein assessed with western blotting (Figure 6D).

Additionally, in our previous study [17], we demonstrated the potential of verteporfin to inhibit SARS-CoV-2 infection, presumably by interfering with the interaction between ACE2 and the receptor-binding domain of viral S protein. Remarkably, within the iRep-N-NLuc-cell model, verteporfin and nirmatrelvir demonstrated IC_50_ values of 0.583 μM and 0.1395 μM, respectively, for impeding NLuc reporter gene activity (Figure 6E,G). This finding suggests that verteporfin could potentially exert additional interference in viral replication processes. Verteporfin cytotoxicity assays with BHK-21 were performed, and the selection index obtained was 14.1 (Figure 6E). Consistent with this finding, verteporfin significantly suppressed the recombinant N sgRNAs production (Figure 6F). Taken together, these results suggested that the iRep-N-NLuc-cell model is practically useful and convenient for the screening and evaluation of antiviral compounds.

## 4. Discussion

The emergence of SARS-CoV-2 represents a significant threat to public health, prompting researchers to focus on establishing a biosafety SARS-CoV-2 replicon model. Such a system can serve as a comprehensive antiviral drug screening platform, facilitating the identification and exploration of potent and safe antiviral agents from diverse compound libraries. Our initial study confirmed the replicative functionality of the Rep-S-EGFP and Rep-S-NLuc constructs, with the Rep-S-NLuc reporter exhibiting relatively low sensitivity. A previous study integrated a recombinant SARS-CoV-2 replicon under the CMV promoter, showing limited sensitivity of the reporter gene [9]. The authors postulated that their system displayed resistance to antiviral medications. In our study, based on the validation results for Rep-N-NLuc, it is speculated that the low sensitivity of Rep-S-NLuc is primarily due to the positioning of the reporter gene in the replicon, as the S gene may have much less expression than that of the N gene. Transcriptome sequencing studies in virus-infected host cells have demonstrated a gradual increase in the expression levels of canonical structural genes and their associated accessory genes along the 5′-3′ RNA of the genome, supporting this hypothesis [21,22]. Furthermore, the background signals inherent to replicon plasmids may represent unrelated signals independent of replication. This phenomenon reportedly originates from mRNA splicing, given that primary replicon mRNA is transcribed from nuclear plasmid DNA [12]. In the case of Rep-S-EGFP, the relative background signal of the EGFP fluorescence is probably lower that of the NLuc-driven bioluminescence, due to their distinctive attributes and detection methods. A disadvantage of Rep-N-NLuc is that the replicon must be rescued by N protein complementation. Nevertheless, this “weakness” could be utilized as a strength of the replicon system for N protein-related virological research. For example, as the Omicron variant shows an increased frequency of N mutations compared with other variants, this system may enable the examination of the effects of these mutations on viral replication capability.

Based on the Rep-N-NLuc construct, we further developed a cell-based inducible replicon model that is a simple, and cost-effective high-throughput screening tool for antiviral compounds. For antiviral screening, a variety of efforts have been made to develop cell models with constitutive viral replication. While some researchers made use of antibiotic-resistant gene recombinant RNA replicon to maintain the viral persistence under selective pressure of antibiotics, they encountered difficulties due to gradually reduced signal intensity of reporter gene products [12]. Using a similar strategy [6,8,23], however, we failed to establish cell lines stably expressing Rep-S-EGFP or Rep-S-NLuc in the early attempts. It is speculated that persistent viral replication may interfere with the host cell translation and other essential cellular activities, resulting in irreversible damage to the replicon-positive cells. In this respect, a noncytopathic replicon BHK-21 cell line of SARS-CoV-2 was recently developed by introducing Nsp1 K164A/H165A mutations [24], while the same strategy did not seem to be useful as reported by others [25]. This discrepancy could be due to the different background of the cell model.

To overcome these challenges, we introduced for the first time a Cre-*LoxP*-mediated inducible SARS-CoV-2 replicon cell model. Upon induction of the replicon cell model using the recombinase Cre, successful viral RNA replication and reporter gene expression, facilitated by N protein rescue, were achieved. Notably, it is speculated that a potent intronic sequence may increase parental RNA expression while having a dominant negative effect on the cryptic RNA splicing [16]. The IC_50_ of remdesivir on reporter product activity was determined to be 0.446 μM, consistent with values observed in the sgRNAs in the Rep-S-EGFP model and the authentic virus infection studies [19]. Using this model, we screened traditional Chinese medicine compounds and found that both forsythoside A and verteporfin exhibited significant antiviral effects with low cytotoxicity. Particularly, the novel antiviral properties of verteporfin warrant further clarification.

This cell model can serve as a platform for high-throughput screening and evaluation of anti-SARS-CoV-2 compounds. Nevertheless, we observed a decrease in the reporter gene signal after 10 generations of continuous passages in the cell culture model, possibly due to the continuous proliferation of resistant cells. In the present study, we took advantage of a PiggyBac transposon strategy for chromosomal integration of the DNA sequence encoding recombinant SARS-CoV-2 replicon. This strategy enables integration of multi-copies of recombinant viral cDNA into the chromosome; however, these integrations are not endowed with adequate hereditary stability after cell division. As the PiggyBac transposon strategy enables multi-copy chromosomal integrations, screening stable monoclonal transfectants for optimization is advisable for the future studies. Compared with viral infection, the model utilized in this study solely reflects the characteristics of viral replication, necessitating validation with authentic virus infection to investigate the mechanism of viral replication.

## 5. Conclusions

In conclusion, we successfully developed and optimized an inducible SARS-CoV-2 replicon cell model, providing enhanced stability for antiviral drug screening and virological mechanistic studies. This robust and effective experimental model may help explore the replication and pathogenesis of SARS-CoV-2. Moreover, this model holds promise for broad applications and screening assessment of anti-SARS-CoV-2 therapeutics.

## Data Availability

The data presented in this study are available upon request from the corresponding author.

## Supplementary Materials

The following supporting information can be downloaded at: https://www.mdpi.com/article/10.3390/v16050708/s1, Figure S1: Protoporphyrin IX does not inhibit Rep-S-EGFP-driven viral replication in BHK-21 cells. BHK-21 cells transfected with Rep-S-EGFP were treated with varying concentrations of protoporphyrin IX for 36 h before qRT-PCR detection. Data represent mean ± SEM from three independent experiments. “ns” indicates no statistically significant difference; Figure S2: Proprietary compounds targeting the viral protease 3CL inhibit Rep-S-EGFP replication. (A) VeroE6 cells were transfected with Rep-S-EGFP and treated with proprietary compounds at indicated doses. After 48 h, N protein expression was determined using western blotting. (B,C) VeroE6 cells were transfected with Rep-S-EGFP and exposed to varying concentrations of the proprietary compounds S6 and S77, as depicted in the figure. The sample processing and detection procedures are identical to those used in (A); Figure S3: Integration site and replication competence validation of inducible expression cassettes in the replicon. (A) An intron was integrated between nt 66 and 67 (i.e., CAG66^G67; ^ indicates the potential exon/exon boundary) or nt 175 and 176 (i.e., AAG175^G176) of the NSP1 gene to construct iRep-N-NLuc-CAGG or iRep-N-NLuc-AAGG. PiggyBac 5′ TR and chicken β-globin insulators are inserted at the 5′ end of the CMV promoter and at the 3′ end of the HDV RZ region, respectively, flanking the transcription unit. (B) BHK-21 cells were co-transfected with iRep-N-NLuc-CAGG or iRep-N-NLuc-AAGG and pcDNA3.1-N, plus a Renilla luciferase normalization plasmid. After transfection for 6 h, the cells were treated with 10 μM remdesivir for 24 h, followed by cell lysis for NLuc activity detection. Data are presented as the mean ± SEM from three independent experiments. The fold change in NLuc activity (relative luminescence units, RLU) was normalized to that of Renilla luciferase activity. ** indicates *p* < 0.01. (C) NLuc activity data of samples iRep-N-NLuc-CAGG or iRep-N-NLuc-AAGG in (B). *** indicates *p* < 0.001.
