# Supplementary figures and images for: Development of a Cell Culture Model for Inducible SARS-CoV-2 Replication
Source: Viruses. 2024 Apr 29;16(5):708. doi: 10.3390/v16050708 (PMC11125939; doi:10.3390/v16050708)

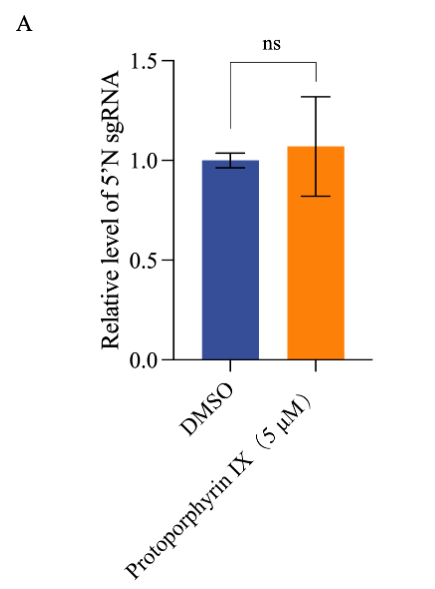

Supplement: Supplementary file 1 [file viruses-16-00708-s001.zip › viruses-2969575 supplementary revised/FS1.png]

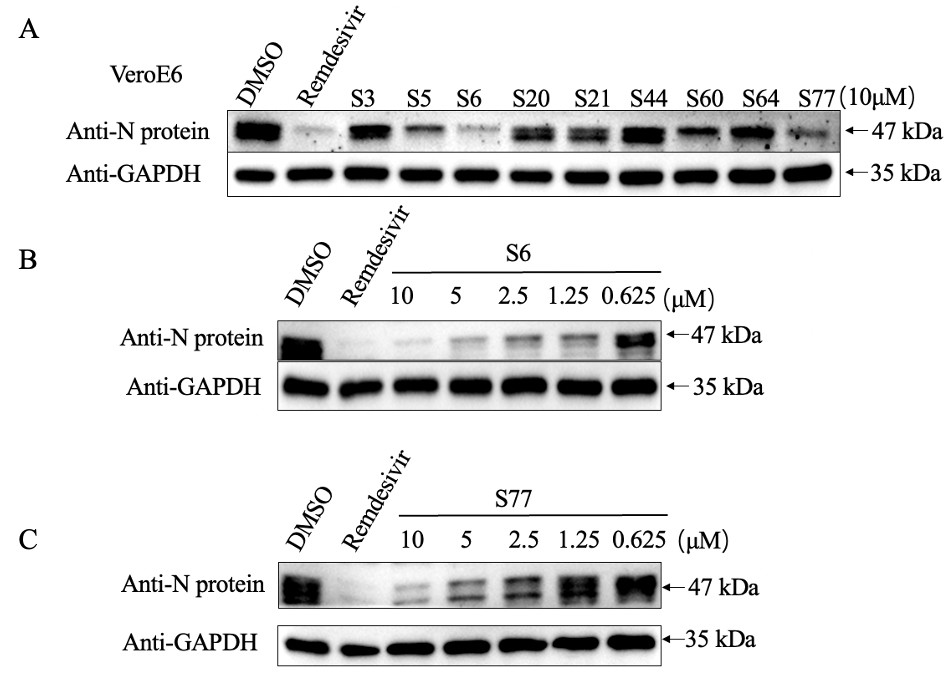

Supplement: Supplementary file 1 [file viruses-16-00708-s001.zip › viruses-2969575 supplementary revised/FS2.png]

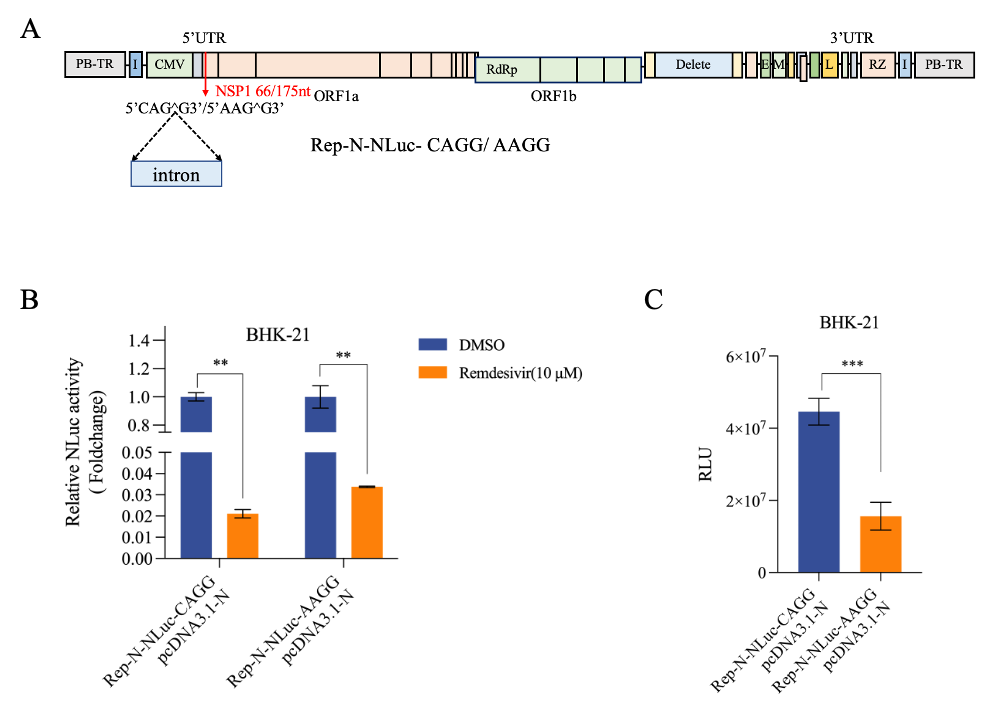

Supplement: Supplementary file 1 [file viruses-16-00708-s001.zip › viruses-2969575 supplementary revised/FS3.png]
